# TCGA RNA-Seq and Tumor-Infiltrating Lymphocyte Imaging Data Reveal Cold Tumor Signatures of Invasive Ductal Carcinomas and Estrogen Receptor-Positive Human Breast Tumors

**DOI:** 10.3390/ijms24119355

**Published:** 2023-05-27

**Authors:** Mayassa J. Bou-Dargham, Linlin Sha, Drishty B. Sarker, Martina Z. Krakora-Compagno, Zhui Chen, Jinfeng Zhang, Qing-Xiang Amy Sang

**Affiliations:** 1 Department of Chemistry and Biochemistry, Florida State University, Tallahassee, FL 32306, USA; mayassa.dargham@pennmedicine.upenn.edu (M.J.B.-D.); ds22@fsu.edu (D.B.S.); 2 Department of Statistics, Florida State University, Tallahassee, FL 32306, USA; ls15w@my.fsu.edu (L.S.); jinfeng@stat.fsu.edu (J.Z.); 3 Institute of Molecular Biophysics, Florida State University, Tallahassee, FL 32306, USA; mkrakora@fsu.edu; 4 Abbisko Therapeutics, Shanghai 200100, China

**Keywords:** immunologically hot tumors, cold tumors, human breast cancer, tumor-infiltrating lymphocytes (TILs), M2 macrophages, immune response, immunotherapy, the cancer genome atlas (TCGA), the cancer imaging archive (TCIA), secondary analysis of gene expression dataset

## Abstract

Comparative studies of immune-active hot and immune-deserted cold tumors are critical for identifying therapeutic targets and strategies to improve immunotherapy outcomes in cancer patients. Tumors with high tumor-infiltrating lymphocytes (TILs) are likely to respond to immunotherapy. We used the human breast cancer RNA-seq data from the cancer genome atlas (TCGA) and classified them into hot and cold tumors based on their lymphocyte infiltration scores. We compared the immune profiles of hot and cold tumors, their corresponding normal tissue adjacent to the tumor (NAT), and normal breast tissues from healthy individuals from the Genotype-Tissue Expression (GTEx) database. Cold tumors showed a significantly lower effector T cells, lower levels of antigen presentation, higher pro-tumorigenic M2 macrophages, and higher expression of extracellular matrix (ECM) stiffness-associated genes. Hot/cold dichotomy was further tested using TIL maps and H&E whole-slide pathology images from the cancer imaging archive (TCIA). Analysis of both datasets revealed that infiltrating ductal carcinoma and estrogen receptor ER-positive tumors were significantly associated with cold features. However, only TIL map analysis indicated lobular carcinomas as cold tumors and triple-negative breast cancers (TNBC) as hot tumors. Thus, RNA-seq data may be clinically relevant to tumor immune signatures when the results are supported by pathological evidence.

## 1. Introduction

Latest cancer research has markedly revolutionized cancer treatments with a shift from conventional therapies, such as chemotherapy and radiotherapy, towards immunotherapy. The first immunotherapy for breast cancer was approved by the US food and drug administration (FDA) in 2019 for advanced triple-negative breast cancer (TNBC). This treatment targets PD-L1 (Tecentriq^®^) in combination with chemotherapy (Abraxane^®^; nab-paclitaxel) [1,2]. Another accelerated approval was given to pembrolizumab in 2020 (KEYTRUDA^®^) in combination with chemotherapy (paclitaxel protein-bound/paclitaxel, or gemcitabine plus carboplatin) for the treatment of patients with locally recurrent unresectable or metastatic TNBC whose tumors express PD-L1 [3]. As anti-PD-1/PD-L1 effectiveness is influenced by PD-L1 status and tumor-infiltrating lymphocytes (TIL) [4], chemotherapy may enhance the release of tumor antigens and lymphocyte infiltration [2]. The cytotoxic effects of chemotherapy may still be avoided by increasing TILs via other methods.

Tumors with high infiltration of lymphocytes are referred to as “immunologically hot” or “T-cell inflamed,” whereas those with low infiltration of lymphocytes are referred to as “immunologically cold” or “non-inflamed” tumors [5]. Several studies highlighted the good prognosis associated with immunologically hot tumors and their better response to treatment than that seen in cold tumors. This was especially observed among patients with high infiltration of CD8+ T cells in several cancers, including breast cancer [6,7,8,9,10]. Although adoptive T cell therapy, namely CAR T cell treatment, represents a good approach to increase the infiltration and activation of T cells against the tumor, the identification of a suitable cancer-specific antigen remains challenging in solid malignancies [11]. Although some hot tumors may not be associated with a good prognosis, they may still benefit from combination therapy that can prevent immune evasion [12]. Thus, one approach would be to understand what hinders immune cell infiltration in cold non-responsive tumors to identify potential ways to improve response to therapy. However, the exact mechanisms by which lymphocyte infiltration is limited in certain breast tumors have not been fully investigated [5,13].

The crosstalk between tumor cells and tumor-associated cells, especially immune cells, can either inhibit or enhance tumor growth. Tumors can alter the tumor immune microenvironment (TIME) and stimulate the recruitment and differentiation of regulatory T cells (T_reg_), myeloid-derived suppressor cells (MDSCs), tumor-associated macrophages (TAM), and M2 macrophages [14,15,16,17]. The role of T_reg_ is to maintain immune peripheral tolerance by inhibiting the overactivation of cytotoxic T lymphocytes (CTL) through the production of IL-10, TGF-β, and the expression of CTLA4, which prevent co-stimulation-induced activation of CTL [18,19]. M2 macrophages, on the other hand, secrete anti-inflammatory cytokines and maintain tissue homeostasis during wound healing [20]. MDSC in the tumor microenvironment produce reactive oxygen species (ROS) and suppress T cell response, promoting tumor angiogenesis, tumor cell invasion, and metastasis [21,22]. Regulators of peripheral tolerance can impair immune cell trafficking and, in extreme cases, lead to the development of immune-privileged sites [12,23]. Thus, we hypothesize that the extracellular matrix in cold tumors hinders the infiltration of immune cells into the tumor core.

Tumors with high levels of TILs are typically associated with better outcomes. Hence, it is imperative to understand what impedes the infiltration of TILs in cold tumors and what acts favorably in the case of hot tumors. Immune gene expression signature has been the most explored method to characterize hot tumors of different origins and predict their response to immune checkpoint inhibitors [24]. Recurrently present immune genes in these signatures include *IFNG*, *CD8A*, *STAT1*, *GZMA*, *CXCL9*, etc. [25,26]. Beyond protein-coding genes, inflammation-related long non-coding RNAs (lncRNAs) can serve as markers to differentiate hot tumors from cold ones in bladder cancer [27], and necroptosis-related lncRNAs can serve the purpose in gastric cancer [28] and cutaneous melanoma [29]. Immune-related differentially expressed miRNAs have also been shown to differentiate between hot and cold tumors [30].

In this study, we used The Cancer Genome Atlas (TCGA) RNA-seq data of breast cancer (BRCA) and their matched normal tissue adjacent to the tumor (NAT) [31]. The tumor specimens were dichotomized into hot and cold tumors based on their lymphocyte scores, as described by Thorsson et al. [32]. Based on the expression data, the corresponding immune cell signatures were derived for TCGA breast tumor and NAT samples, and for the normal breast tissue samples from healthy individuals obtained from the Genotype-Tissue Expression (GTEx) database [33]. The differences in gene expression and immune pathways between hot and cold breast cancers were identified using the data to understand the signatures of these two distinct tumor types. We then justified the RNA-seq-based hot/cold classification by utilizing The Cancer Imaging Archive (TCIA) analysis results of pathology image-derived TIL maps [34], as performed by Saltz et al. [35]. Altogether, we tested the merit of RNA-seq data analysis to characterize immune responses of hot and cold tumors and compared the RNA-seq-based approach to TIL map-based hot/cold status assignment.

## 2. Results

### 2.1. Grouping Breast Tumor Samples into Hot and Cold Groups Based on Lymphocyte Scores

Tumors are considered highly immunogenic when characterized by high infiltration of lymphocytes. We used the lymphocyte scoring proposed by Thorsson et al. to dichotomize TCGA breast cancer samples into hot and cold tumors [32]. The lymphocyte score uses the abundance data provided by CIBERSORT [36] and is the sum of the abundance data of CD8+, CD4+, NK, and B cells. Samples with a score less than 0.45 were categorized as cold tumors and those greater than 0.45 as hot tumors (Supplementary File S2).

**Figure 1:**
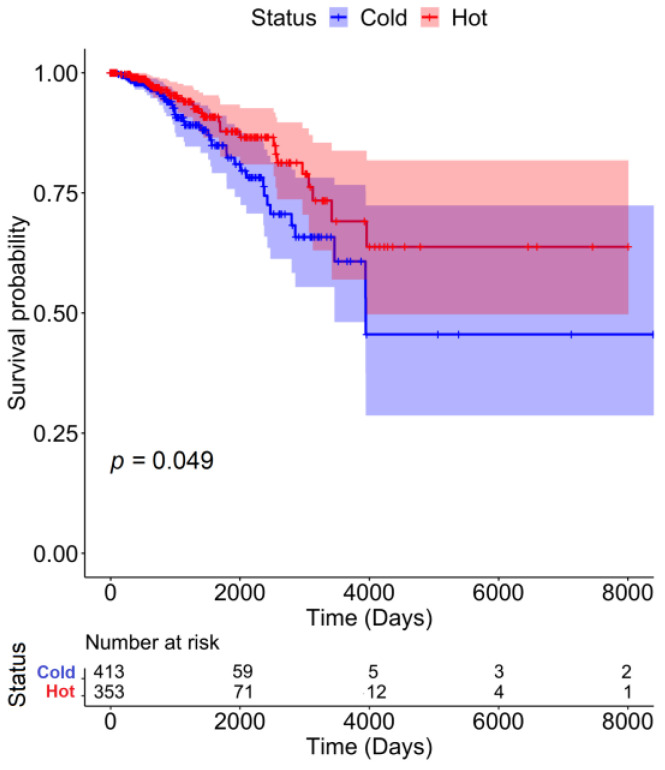
Patients with Hot Tumors Show Higher Survival than Those with Cold Tumors. Survival curves were estimated using the Kaplan–Meier method and statistical comparison was done using the log-rank test.

**Figure 2:**
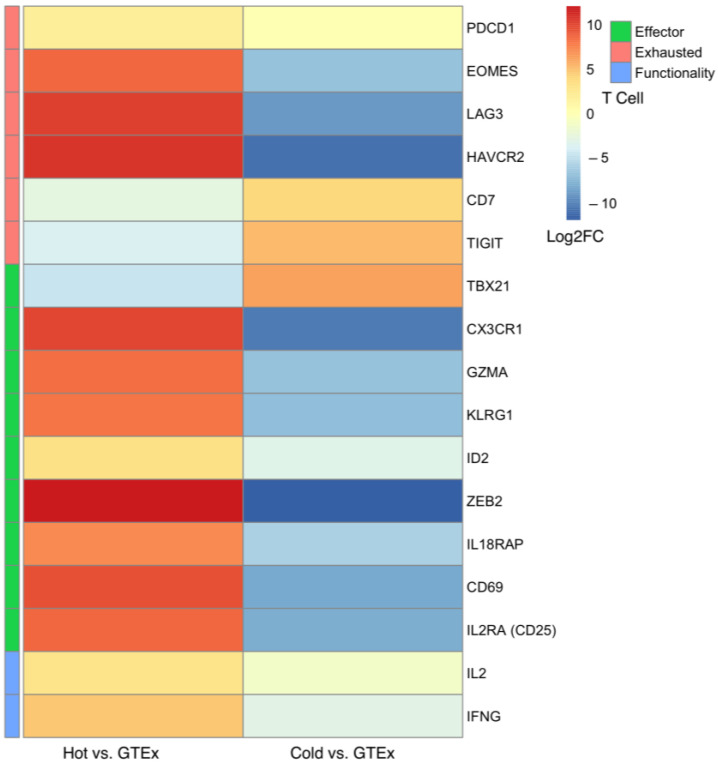
DESeq2 Results for Differentially Expressed Genes Involved in Immune Activation and Evasion in Comparison Analysis of Cold or Hot Tumors with Normal GTEx. The genes are classified based on their implication in T cell states–effector, exhausted, and functionality. Programmed cell death protein 1 (*PDCD1*), eomesodermin (*EOMES*), lymphocyte activating 3 (*LAG3*), hepatitis A virus cellular receptor 2 (*HAVCR2*), CD7 molecule (*CD7*), T cell immunoreceptor with Ig and ITIM domains (*TIGIT*), T-box transcription factor 21 (*TBX21*), C-X3-C motif chemokine receptor 1 (*CX3CR1*), granzyme A (*GZMA*), killer cell lectin like receptor G1 (*KLRG1*), inhibitor of DNA binding 2 (*ID2*), zinc finger E-box binding homeobox 2 (*ZEB2*), interleukin 18 receptor accessory protein (*IL18RAP*), CD69 molecule (*CD69*), interleukin 2 receptor subunit alpha (*IL2RA*), interleukin 2 (*IL2*), interferon gamma (*IFNG*).

**Figure 3:**
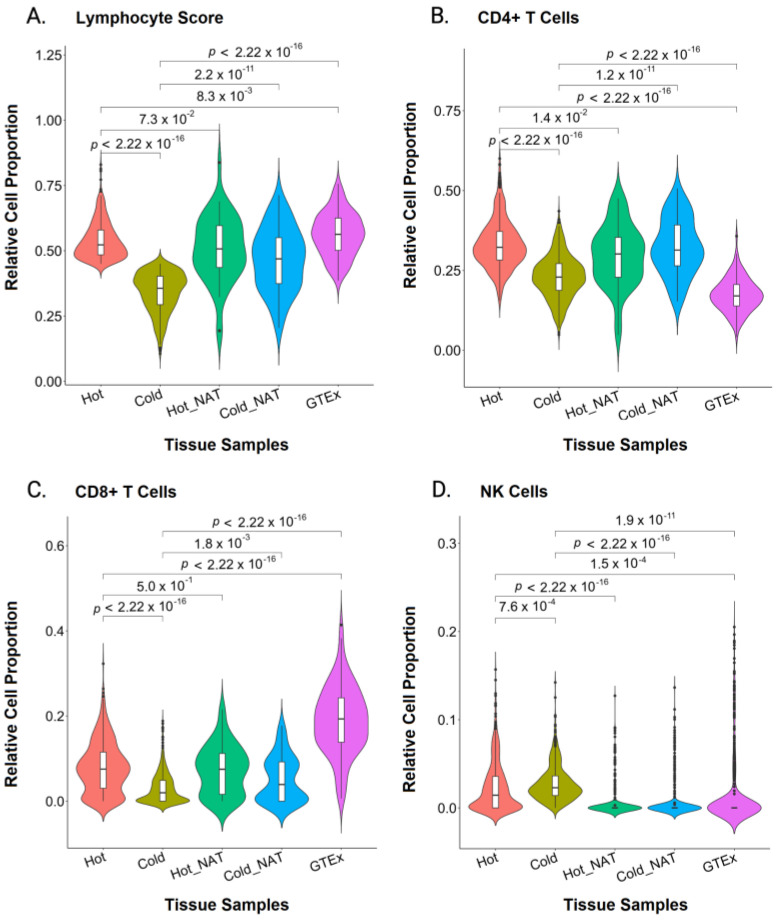
Violin Plot Representations of Immune Cell Abundance Distribution. Hot and Cold Tumors, Normal Tissue Adjacent to Hot and Cold Tumors (Hot NAT and Cold NAT, respectively), and Genotype-Tissue Expression (GTEx) Normal Tissue were analyzed for total lymphocyte score (**A**), CD8+ T cells (**B**), CD4+ T cells (**C**), and natural killer (NK) cells (**D**). The statistical analysis was done using a pairwise *t*-test and *p* < 0.05 indicates statistical significance.

**Figure 4:**
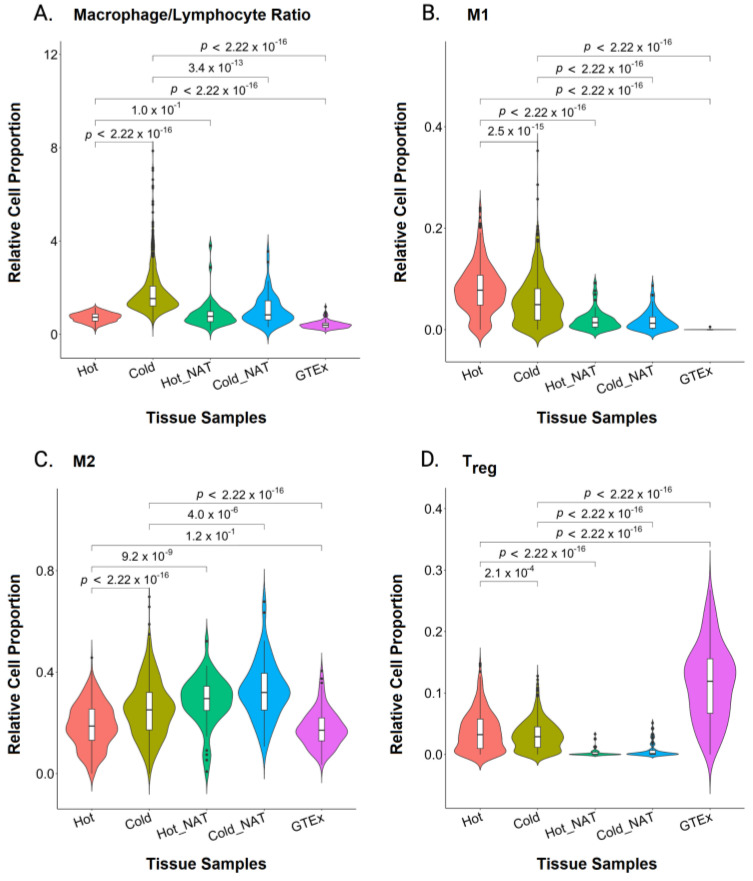
Violin Plot Representations of the Abundance Distribution of Macrophage and Regulatory T cells. Hot and cold tumors, normal tissue adjacent to hot and cold tumors (Hot NAT and Cold NAT, respectively), and Genotype-Tissue Expression (GTEx) normal tissue were analyzed for macrophage-to-lymphocyte ratio (**A**), M1 macrophages (**B**), M2 macrophages (**C**), and regulatory T cells (T_reg_) (**D**). The statistical analysis was done using a pairwise *t*-test and *p* < 0.05 indicates statistical significance.

As expected, patients with hot tumors showed significantly higher survival than that of the cold tumor group (Figure 1). Hot tumors showed a higher effector CD8+ T cell activation, whereas cold tumors had significantly lower effector T cells (Figure 2). Although normal GTEx samples had a higher average lymphocyte score than the lymphocyte score of hot and cold tumors (Figure 3A), namely, higher CD8+ T (Figure 3C) and T_reg_ cells (Figure 4D), gene expression analysis showed that the CD8+ T cells may not be as active (Figure 2). Furthermore, pathway analysis showed that immune cell recruitment and activation in hot tumors were significantly more enriched than immune cell enrichment and activation in cold and normal GTEx samples (Supplementary Files S7 and S8). In addition, we investigated differential expression of transcription factors between hot and cold tumors. We selected the top 100 differentially expressed genes between hot and cold tumors and then identified transcription factors using DAVID (Supplementary File S9). Though it is not feasible to determine which transcription factors are expressed in immune cells or tumor cells based on bulk RNA-seq data, we found eomesodermin (EOMES, important for effector T cell function) and Spi-B transcription factor (SPIB, promotes pDC and IFN producing cells) among the upregulated transcription factors.

### 2.2. Cold NAT and Hot NAT Are Immunologically Active but Cold Tumors Are Immunologically Inactive

**Figure 5:**
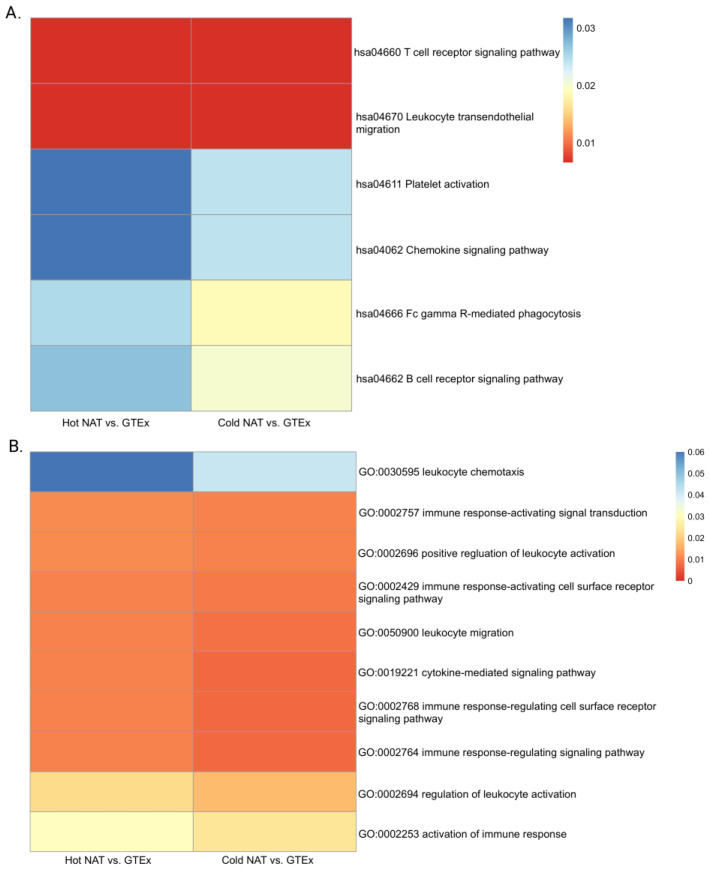
Activated Leukocyte Migration in Immune Pathway Analysis of Differentially Expressed Genes in Comparisons of Hot or Cold NAT with GTEx. Summary from KEGG (**A**) and GO (**B**) presented.

Hot NAT and cold NAT showed upregulated immune pathways compared to those in normal tissues, specifically those involved in leukocyte migration and activation, chemokines, and chemotaxis (Figure 5). The KEGG and GO pathways and processes for cold tumors showed no significant difference from those of the normal GTEx samples (Supplementary Files S7 and S8). Thus, unlike hot tumors, where both the tumor and the hot NAT samples were immunologically active, cold NAT samples were more immunologically active than GTEx or the cold tumors themselves (Supplementary Files S7 and S8).

### 2.3. High M2 Macrophages in Cold Tumors

To better understand the immune profile in hot and cold tumors, we checked the abundance of immune cells in hot samples, cold samples, their corresponding NAT samples, and normal GTEx samples (Supplementary File S1).

Cold tumors showed the lowest lymphocyte score and CD8+ T cell abundance (Figure 3A,C), despite their slightly higher NK cell abundance (Figure 3D). CD4+ cells in cold tumors were second to lowest after GTEx (Figure 3B). To understand the differences between the tumor samples and their NAT samples better, we compared each tumor sample to its corresponding NAT sample isolated from the same patient. The cold NAT samples showed an overall higher lymphocyte score than the lymphocyte score of their corresponding cold tumors (Supplementary File S10). This lower lymphocyte score in cold tumors suggests either a potential “barrier” that is decreasing the infiltration of lymphocytes into the tumor core or a highly suppressive environment in the tumor samples.

To understand the cause of this reduced lymphocyte infiltration, we measured the inflammatory (macrophages) and cytotoxic (lymphocyte) activities. The macrophage-to-lymphocyte ratio in cold tumors was higher than the ratio observed in hot tumors, indicating a lower cytotoxic to inflammatory immune response (Figure 4A). To understand which macrophage phenotype was more prevalent, we compared the inflammatory M1 macrophage and anti-inflammatory M2 macrophage abundance (Figure 4B,C). Cold tumors showed a significantly higher M2 abundance than the M2 abundance seen in both hot tumors and GTEx. However, M2 abundance in both hot and cold NAT was significantly higher than the M2 abundance seen in hot and cold tumors. This suggests high M2 macrophage recruitment to tissues adjacent to tumors. Furthermore, our data demonstrate the highest T_reg_ count in GTEx samples and a higher tumor abundance in hot samples compared to cold (Figure 4D).

### 2.4. Genes Correlated with Low Lymphocyte Score in Cold Tumors Reveal Low Antigen Presentation and Increased Matrix Remodeling

**Figure 6:**
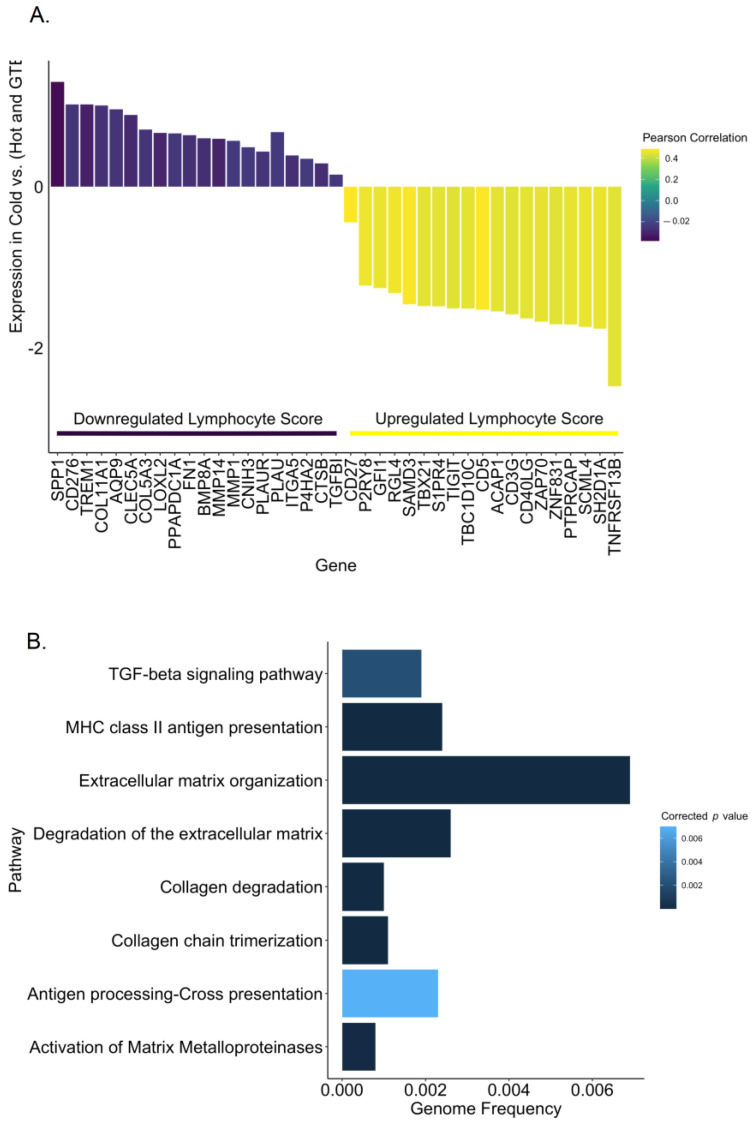
Upregulated Genes in Cold Tumors are Associated with a Low Lymphocyte Score and Weak Lymphocyte Recruitment. Overlapping upregulated or downregulated genes between (cold vs. hot) and (cold vs. GTEx) and their correlation with the lymphocyte score (**A**); Pathway analysis for the upregulated overlapping genes (**B**).

When we compared cold tumor samples with hot tumor samples and with GTEx normal samples, we identified the differentially expressed genes that overlapped in both comparisons. To identify the genes contributing to the low infiltration of lymphocytes in cold tumors, we looked at the correlation between the lymphocyte score and the overlapping differentially expressed genes (Supplementary File S11). As expected, the list of overlapping genes upregulated in cold tumors correlated with a negative lymphocyte score, while the downregulated genes correlated with a positive lymphocyte score (Figure 6A, Supplementary Files S4 and S12). Although the correlation was not too strong, the pathway analysis revealed weak recruitment of lymphocytes in cold tumors (low chemokines and cytokines), lower antigen presentation yet higher inflammation, activation of MMPs, and matrix remodeling (Figure 6B, Supplementary File S5).

### 2.5. Infiltrating Ductal Carcinoma and ER-Positive Tumors Exhibit Cold Tumor Signiatures

To investigate the association of tumor histology and receptor status with the RNA-seq-based hot/cold status, we did Fisher’s exact tests. The results showed no significant association between HER2 or TNBC status with hot or cold status. However, infiltrating lobular carcinoma showed significant association with hot tumors. ER-positive tumors, infiltrating ductal carcinoma, and mucinous carcinoma were associated with cold tumors (Table 1 and Supplementary File S6). As ER-negative samples were split equally between hot and cold tumors, their assignment to either of the statuses was inconclusive.

### 2.6. RNA-Seq-Based Hot/Cold Classification and Pathological TIL Patterns Mostly Coincide

For all the breast tumor histology types reported in TIL map dataset, we analyzed the TIL map structural patterns (Supplementary File S16). Fisher’s exact test was performed to find any association between TIL pattern-based hot/cold status and tumor histology (Supplementary File S6). Lobular carcinoma, infiltrating ductal carcinoma, and ER-positive samples were significantly associated with cold tumors, whereas TNBC samples showed a significant association with hot tumors. Infiltrating ductal and lobular carcinoma, mucinous carcinoma, and HER2 status could not be associated with any status (Table 2).

The concordance of RNA-seq- and TIL map-based approaches to hot/cold tumor dichotomy is summarized in Table 3.

## 3. Discussion

Tumor-infiltrating lymphocytes (TIL) are vital to the immune response of breast cancer. TILs were found to be a positive prognostic biomarker in non-luminal subtypes [37]; however, the predictive value of TILs in estrogen receptor ER-positive breast tumors is less clear compared to other subtypes [38]. This study analyzed the TIL abundance in human breast tumor subtypes using TCGA RNA-seq and TCIA pathological imaging data.

Although RNA-seq data analysis suggests that infiltrating lobular carcinoma is correlated with hot tumors, the TIL map analysis demonstrated its association with cold tumor, which is consistent with previous scientific findings [12,39,40]. The TIL dataset suggested that infiltrating ductal carcinoma tend to be cold tumors, consistent with its statistically significant association with the same status based on RNA-seq analysis. Studies have reported frequent, increased lymphocyte infiltration into the stroma of ductal carcinomas but lower intratumoral TIL [41]. TIL map-based hot/cold assignment was inconclusive for mucinous carcinoma, likely due to the small sample size. Interestingly, the RNA-seq analysis identified mucinous carcinoma as a cold tumor. This result is consistent with pathological evidence that show mucinous carcinoma is typically TIL-depleted [42,43].

RNA-seq-based analysis conformed to the TIL map finding for HER2-positive cases being neither hot nor cold. Although the RNA-seq analysis showed no significant association between TNBC and hot or cold status, the TIL dataset led us to identify TNBC as a hot tumor. The immunogenic nature of TNBC is well supported in the literature [44,45]. Finally, ER-positive tumors were associated with the cold status in both RNA-seq and TIL-pattern analysis. The suggested coldness of ER-positive tumors is also consistent with the literature [41,46,47]. Overall, our findings imply that RNA-seq-based immunogenicity prediction may conform to TIL map-based findings in certain breast tumor subtypes and may have prognostic value for clinical decision-making if validated by pathological evidence. Since our cancer data are solely based on bulk RNA-seq data from TCGA, future single-cell RNA-seq and protein expression analyses may further verify our findings. TCGA breast cancer data mostly contain ER-positive specimens because a majority of the breast cancer population are ER-positive [48]. Thus, future studies focusing on other subtypes would deepen our understanding of their immune profiles.

We found that low antigen presentation and abundance of M2 macrophages contribute to immunologically cold breast cancer. In contrast, the overall lymphocyte score is high in cold NAT samples. This suggests that ECM stiffness and M2 macrophages may be preventing lymphocyte infiltration to the tumor core in cold tumors. This phenomenon was reported previously in other cancers, and such sites were referred to as “immune privileged” [49,50,51]. Future experiments are needed to show that M2 macrophages inhibit T cell infiltration in vitro or in vivo in human breast cancer.

Tumor-associated macrophages (TAM) and M2 macrophages in the tumor microenvironment (TME) increase tumor malignancy, alter the activation of macrophages, induce immunosuppression, and trigger lymphocyte apoptosis [21,22,52,53,54]. It has been previously shown that CD8+ T cells infiltrating pancreatic cancer were mainly found in the fibrous stroma away from the cancer cells [55], and macrophages were found to regulate the infiltration of T cells into the tumor core, establishing a site of immune privilege in pancreatic carcinoma [56]. Furthermore, the abundance of M2 was associated with reduced patient survival [52,57]. Thus, M2 macrophages represent a prognostic factor in breast cancer, when targeted, TILs are expected to increase.

The high levels of M2 macrophages in benign NAT tissues might be a part of the wound healing process to maintain tissue homeostasis and tissue repair [20]. The presence of NAT in the vicinity of the tumor requires meticulous immune control as they may represent field cancerization [58]. Field cancerization can be initiated and propagated in many different ways, including chronic inflammation, mutagens, and reactive oxygen species (ROS) [20,59]. The lymphocyte scores that are similar to those observed in hot tumors and the high recruitment of leukocytes suggest that NAT tissues are immunologically active. Therefore, NAT tissues may not be a valid normal reference for immune infiltration in cancer studies. To overcome this limitation, we have used GTEx breast specimens as normal control. The applicability of GTEx samples for comparison with tumor specimens is endorsed by this statement that describes the project—“Of course, not all organs will be entirely normal, but donor eligibility is broad and is not restricted to specific diseases or conditions, and it is expected that many organs will be free of major disease processes.” [60].

Immune cells are a consistent part of the normal breast tissue where lobules constitute the primary site of immune cell localization. The non-lactating normal breast tissue is characterized by a mucosal immune response manifested by CD8+ and dendritic cells integrated within the breast epithelium [61]. Peripheral tolerance is maintained by an immunosuppressive microenvironment manifested by the high abundance of tissue-resident T_reg_s [16,62]. Our investigation showed that tumor abundance of CD8+ lymphocytes was lower than the CD8+ lymphocytes seen in normal tissues. However, gene and cytokine expression data indicate the CD8+ lymphocytes are not as active in normal GTEx samples. The low abundance of CD8+ lymphocytes in tumor samples may be due to immune checkpoint inhibition as indicated by the high expression of PD-1, PD-L2, and CTLA4 [63,64,65].

In hot tumors, T cell immune checkpoint inhibition and T_reg_ suppression could be treated by targeting the involved immunosuppressive molecules such as CTLA4 and PD-1 [16]. Cold tumors, on the other hand, may be treated by targeting M2 macrophages and adoptive T cell therapy to overcome the low immunogenicity and facilitate T cell infiltration [5,66,67,68]. Understanding the immune characteristics of breast tumors may help determine the choice of immunotherapy to target tumors in a potent and efficient manner.

## 4. Materials and Methods

### 4.1. Expression Data of Breast Cancer, Normal Tissue Adjacent to Tumor (NAT), and Normal Healthy Tissue Samples

RNA-seq data of 1082 female breast cancer (BRCA) samples were obtained from TCGA (https://www.cancer.gov (accessed on 24 May 2023); Legacy Archive hg19 data) together with those of 112 normal tissue adjacent to tumor (NAT) tissue samples. Normal healthy breast RNA-seq expression data for 115 samples were obtained from GTEx (https://www.gtexportal.org (accessed on 24 May 2023)), the version was v7. We generated three data matrices: a cancer matrix (19,703 × 1082), a normal tissue adjacent to the tumor (NAT) matrix (19,703 × 112), and a normal matrix (19,703 × 115). To ensure the quality and consistency of data, 19,703 overlapping genes between TCGA and GTEx databases were selected. The clinical information of the corresponding patients was obtained from TCGA and GTEx databases.

### 4.2. Immune Cell Analysis and Hot/Cold Status Assignment

CIBERSORT was used to estimate the immune cell composition of the tumor and normal breast tissue samples. A lymphocyte score was calculated by adding the scores of individual lymphocyte populations (B cells, T cells, and NK cells), as described by Thorsson et al. (Supplementary File S1) [32]. The cancer samples were then dichotomized into hot and cold groups based on their lymphocyte scores: ≤0.45 for cold tumors and >0.45 for hot tumors (Supplementary File S2). The cutoff that was used was based on the score distribution across all samples. We tested 0.4, 0.45, and 0.5, and decided to use 0.45 as it resulted in more differentially expressed genes between the hot and cold groups (Supplementary File S2). NAT samples adjacent to hot tumors were labeled hot NAT and those adjacent to cold tumors were termed cold NAT. The data matrices used are summarized in Table 4. Violin plots displaying immune cell distribution were generated using the *ggplot2* package in R [69].

### 4.3. Differential Gene Expression Analysis

Differential gene expression analysis between any two groups was done using the DESeq2 package in R (Supplementary File S3) [70]. A gene was considered to be differentially expressed between groups when the adjusted *p* value was less than 0.05 and the log2 fold change was greater than 2. Genes with a log2 fold change of less than 2 yet with a significant *p* value were considered minimally differentially expressed. The overlapping upregulated and downregulated genes from the comparisons between cold tumors and either hot tumors or GTEx samples were checked for their correlation with the lymphocyte score using the Pearson product-moment correlation (Supplementary File S4).

### 4.4. Pathway Analysis

To identify significantly enriched processes and pathways, we did an enrichment analysis on immune-related pathways from KEGG and GO terms using the Bioconductor packages Pathview and Gage in R [71,72]. For the significantly upregulated and downregulated overlapping genes in the comparisons between cold tumors and either hot tumors or GTEx samples, we used the Comparative Toxicogenomics Database (CTD) set analyzer (http://ctdbase.org (accessed on 24 May 2023)) (Supplementary File S5). DAVID (https://david.ncifcrf.gov/ (accessed on 24 May 2023)) was used to identify transcription factors. T effector, T exhausted, and T cell functionality genes were manually curated based on the T cell literature [73,74].

### 4.5. Verification of RNA-Seq-Based Tumor Dichotomy Using Matched Pathology Images

RNA-seq-based assignment of breast tumor types to either hot or cold category was further tested against tumor-infiltrating lymphocyte maps from TCGA and H&E whole-slide pathology images under TCIA [34]. The dataset contained TIL patterns in the breast tumors of 920 patients of the same TCGA cohort we analyzed RNA-seq data for (Supplementary File S13). Out of 920, we could link 915 patient IDs to the existing RNA-seq dataset, enabling the verification of RNA-seq-based dichotomy (Supplementary File S14). The distribution matrix of TCGA RNA-seq histology and TIL map histology was constructed as well (Supplementary File S15). Distinct TIL structural patterns, as described by Saltz et al., were translated to hot/cold tumor features for verification study [35]. The “Brisk, diffuse” pattern was considered a hot tumor feature for having >30% TILs in the intratumoral component. Contrarily, “None” and “Non-brisk, focal” structural patterns were adjudged the hallmarks of cold tumors since they had <5% TILs. “Brisk, band-like” and “Non-brisk, multi-focal” were included neither in the hot nor cold category to avoid ambiguity [35].

### 4.6. Fisher’s Exact Test

To investigate whether certain clinical characteristics would associate with the immunological coldness or hotness of breast tumors, we conducted Fisher’s exact tests in R. The *p* values were calculated by comparing the distribution of samples in the hot and cold groups for each category to the distribution of remaining samples in these two groups (Supplementary File S6 and Table 1 and Table 2). A cutoff *p* value of 0.05 was used to determine statistical significance.

### 4.7. Survival Analysis

Survival curves were estimated using the Kaplan–Meier method and the statistical comparison was done using the log-rank test. The log-rank test is used to test the null hypothesis of no difference in survival between two or more independent groups.

## 5. Conclusions

In summary, this study analyzes the immune microenvironment in hot and cold breast tumors, their adjacent normal tissues, and normal healthy breast tissues based on TCGA RNA-seq and GTEx data. The hot/cold assignment of breast tumors was verified using pathology image-derived TIL maps. We demonstrated that infiltrating ductal carcinoma and estrogen receptor ER-positive tumors were significantly associated with cold phenotypes. However, RNA-seq results should be interpreted with caution and accompanied with pathological evidence for meaningful clinical decision-making.

We provided evidence for high levels of pro-tumorigenic M2 macrophages in cold tumors and showed that T cell suppression is the main immune evasion mechanism in hot breast tumors. We hypothesize that elevated levels of M2 macrophages and ECM rigidity may restrict lymphocyte infiltration into the cold tumor core. Our findings also indicate M2 macrophages are a promising immunotherapeutic target in human breast cancer.

## Figures and Tables

**Table 1:** The Association of Tumor Histology and Receptor Status with Immunologically Hot and Cold Tumors Based on RNA-seq Data.

Histology/Receptor Status	Cold Samples	Hot Samples	Total Samples	*p* Value *
Infiltrating Lobular Carcinoma	89 (44.50%)	111 (55.50%)	200	**6.34 × 10^−4^**
Infiltrating Ductal Carcinoma	462 (60%)	308 (40.00%)	770	**1.68 × 10^−2^**
Mucinous Carcinoma	16 (94.12%)	1 (5.88%)	17	**1.91 × 10^−3^**
ER-positive	475 (59.97%)	317 (40.03%)	792	**1.23 × 10^−2^**
ER-negative	119 (50.64%)	116 (49.36%)	235	
HER2-positive	97 (60.25%)	64 (39.75%)	161	7.85 × 10^−1^
HER2-negative	324 (58.80%)	227 (41.20%)	551	
TNBC	61 (54.46%)	51 (45.54%)	112	4.42 × 10^−1^

* *p* values for infiltrating lobular carcinoma, infiltrating ductal carcinoma, mucinous carcinoma, and TNBC were calculated by comparing the hot/cold distributions of these individual groups to that of the remaining samples.

**Table 2:** The Association of Tumor Histology with Immunologically Hot and Cold Tumors Based on TIL Map.

Histology/Receptor Status	Cold Samples ^a^	Hot Samples ^b^	Total Samples	*p* Value *
Lobular Carcinoma	87 (74.36%)	30 (25.64%)	117	**2.91 × 10^−3^**
Infiltrating Ductal Carcinoma	239 (58.01%)	173 (41.99%)	412	**7.65 × 10^−4^**
Infiltrating Ductal and Lobular Carcinoma	12 (54.55%)	10 (45.45%)	22	5.02 × 10^−1^
Mucinous Carcinoma	8 (88.89%)	1 (11.11%)	9	1.64 × 10^−1^
ER-positive	318 (67.52%)	153 (32.48%)	471	**1.97 × 10^−6^**
ER-negative	57 (44.19%)	72 (55.81%)	129	
HER2-positive	52 (58.43%)	37 (41.57%)	89	4.08 × 10^−1^
HER2-negative	323 (63.21%)	188 (36.79%)	511	
TNBC	40 (40.40%)	59 (59.60%)	99	**1.31 × 10^−6^**

^a^ Cold samples had “None” and “Non-brisk, focal” TIL structural patterns (<5% of the tumors). ^b^ Hot samples had “Brisk, diffuse” TIL patterns (>30% of the tumors). * *p* values for lobular carcinoma, infiltrating ductal carcinoma, infiltrating ductal and lobular carcinoma, mucinous carcinoma, and TNBC were calculated by comparing the hot/cold distributions of these individual groups to that of the remaining samples.

**Table 3:** Summary of the Association Studies between Histology/Receptor Status and Hot/Cold Status via RNA-seq- and TIL Map-based Approaches.

Histology/Receptor Status	RNA-Seq	TIL Map
Infiltrating Lobular Carcinoma/Lobular Carcinoma	**Significant** (Cold 44.50%, **Hot 55.50%)**	**Significant** (**Cold 74.36%**, Hot 25.64%)
Infiltrating Ductal Carcinoma	**Significant** (**Cold 60%**, Hot 40%)	**Significant** (**Cold 58.01%**, Hot 41.99%)
Mucinous Carcinoma	**Significant** (**Cold 94.12%**, Hot 5.88%)	**Non-Significant** (**Cold 88.89%**, Hot 11.11%)
ER	**Significant** (pos.: **Cold 59.97%**, Hot 40.03%) (neg.: **Cold 50.64%**, Hot 49.36%)	**Significant** (pos.: **Cold 67.52%**, Hot 32.48%) (neg.: Cold 44.19%, **Hot 55.81%**)
HER2	**Non-Significant** (pos.: **Cold 60.25%**, Hot 39.75%) (neg.: **Cold 58.80%**, Hot 41.20%)	**Non-Significant** (pos.: **Cold 58.43%**, Hot 41.57%) (neg.: **Cold 63.21%**, Hot 36.79%)
TNBC	**Non-Significant** (**Cold 54.46%**, Hot 45.54%)	**Significant** (Cold 40.40%, **Hot 59.60%**)

**Table 4:** Data Structure and the Number of Samples in Each Group.

**TCGA (1194)**	**TCGA Cancer Samples (1082)**	Cold Tumor Samples (627)
		Hot Tumor Samples (455)
	**TCGA NAT Samples (112)**	Cold NAT Samples (62)
		Hot NAT Samples (50)
**GTEx (115)**	**GTEx Normal (115)**	GTEx Normal (115)

## Data Availability

The data used were obtained from the Cancer Genome Atlas and the Genotype-Tissue Expression databases. All analyses are available in the Supplementary Files.

## Supplementary Materials

The following supporting information can be downloaded at: https://www.mdpi.com/article/10.3390/ijms24119355/s1.
